# Multimodal imaging of apophyseal avulsion fractures in adolescent soccer players: contributions of plain radiography, power Doppler, and magnetic resonance imaging

**DOI:** 10.1590/0100-3984.2025.0059

**Published:** 2025-12-08

**Authors:** Roberto Mogami, Claudio Henrique Ivo de Araújo Ribeiro Filho, Eduardo Brown Guedes dos Santos, André de Almeida Vieira, Lucas Nascimento da Luz, Caio Leal Leidersnaider, Natália Fernandes de Azevedo, Edson Marchiori, Paulo Sérgio Chagas Gomes

**Affiliations:** 1 Programa de Pós-graduação em Ciências Médicas (PGCM) da Universidade do Estado do Rio de Janeiro (UERJ), Rio de Janeiro, RJ, Brazil; 2 Hospital Universitário Pedro Ernesto (HUPE-UERJ), Rio de Janeiro, RJ, Brazil; 3 Vasco da Gama Sociedade Anônima do Futebol, Rio de Janeiro, RJ, Brazil; 4 Programa de Pós-graduação em Radiologia da Universidade Federal do Rio de Janeiro (UFRJ), Rio de Janeiro, RJ, Brazil; 5 Suprema - Faculdade de Ciências Médicas de Três Rios (FCM/TR), Três Rios, RJ, Brazil; 6 Instituto de Educação Física e Desportos (IEFD/UERJ), Rio de Janeiro RJ, Brazil

**Keywords:** Radiography, Magnetic resonance imaging, Fractures, avulsion, Ultrasonography, Doppler, Soccer/injuries., Radiografia, Ressonância magnética, Fratura/avulsão, Ultrassonografia Doppler, Futebol/lesões.

## Abstract

**Objective:**

To evaluate, primarily, the accuracy of plain radiography (X-ray) in diagnosing apophyseal avulsion fractures in adolescent soccer players, using magnetic resonance imaging (MRI) as the gold standard. As secondary objectives, we investigated associations between findings on X-ray, MRI, and power Doppler, as well as the imaging features that distinguish avulsion fractures from apophysitis.

**Materials and Methods:**

This was an observational cross-sectional study involving 33 male athletes 9-17 years of age with clinical suspicion of an apophyseal avulsion fracture. Imaging examinations were performed within the first 24 h after the trauma. We evaluated diagnostic reproducibility among readers, the accuracy of X-ray compared with MRI, and the associations between findings from different imaging methods.

**Results:**

We found that X-ray had an accuracy of 56.0%, with high specificity (71.4%) and positive predictive value (81.8%), although its sensitivity and negative predictive value were relatively low (50.0% and 35.7%, respectively). The power Doppler result was significantly associated with avulsion fractures detected on MRI (*p* = 0.0144). Avulsion fractures were associated with periphyseal edema and intermuscular fluid collections, while apophysitis was associated with bone marrow edema.

**Conclusion:**

X-ray is useful for confirming, but not for excluding, avulsion fractures. Power Doppler and MRI contribute to the differential diagnosis.

## Abstract (pt)

**Objetivo:**

Avaliar, como objetivo primário, a acurácia da radiografia simples no diagnóstico de fraturas avulsivas apofisárias em atletas adolescentes de futebol, usando a ressonância magnética (RM) como padrão ouro. Como objetivos secundários, investigar a associação entre os achados na radiografia simples, RM e *power* Doppler, além de distinguir os achados entre fraturas avulsivas e apofisites.

**Materiais e Métodos:**

Estudo observacional e transversal realizado com 33 atletas do sexo masculino, com idades entre 9 e 17 anos, com suspeita clínica de fratura avulsiva apofisária. Os exames foram realizados em até 24 horas após o trauma. Avaliaramse a reprodutibilidade diagnóstica entre leitores, a acurácia da radiografia simples em relação à RM e as associações entre os diferentes métodos de imagem.

**Resultados:**

A radiografia simples apresentou acurácia de 56%, com alta especificidade (71,4%) e valor preditivo positivo (81,8%), mas sensibilidade (50,0%) e valor preditivo negativo (35,7%) reduzidos. O *power* Doppler associou-se significativamente às fraturas avulsivas na RM (*p* = 0,0144). As fraturas avulsivas foram associadas ao edema periapofisário e coleções intermusculares, e as apofisites, ao edema ósseo.

**Conclusão:**

A radiografia simples é útil para confirmar, mas não para excluir fraturas avulsivas. O Doppler e a RM auxiliam no diagnóstico diferencial.

## INTRODUCTION

Soccer is a widely popular sport, with approximately 240 million players globally and approximately 200,000 athletes competing at the professional level. Among these athletes, adolescents are particularly susceptible to injuries, especially apophyseal avulsion fractures and apophysitis^**(^1^,^2^)**^.

The growth plate (physis) in adolescents is two to five times more fragile than the surrounding fibrous structures like ligaments, capsules, and tendons. Apophyses are secondary ossification centers that do not contribute to longitudinal growth; instead, they serve as attachment points for tendons and are especially vulnerable to traction-related injuries^**(^1^-^3^)**^.

In adolescents, trauma from soccer is often misdiagnosed as muscle strain, which complicates the clinical diagnosis of apophyseal injuries^**(^2^)**^. These injuries can be categorized into two main types: avulsion fractures and apophysitis. Avulsion fractures occur due to acute trauma, resulting in the separation of the apophysis, typically accompanied by changes in the surrounding soft tissues. In contrast, apophysitis is caused by repetitive microtrauma, leading to chronic inflammation of the growth plate cartilage^**(^1^)**^.

Among young soccer players, apophyseal injuries account for 5.1-13.5% of all musculoskeletal injuries, with apophysitis being more prevalent (accounting for up to 90.6% of cases). The anterior inferior iliac spine is the most commonly affected site, accounting for up to 43.3% of all such pelvic injuries^**(^1^)**^.

Avulsion fractures can be diagnosed by using various imaging techniques^**(^1^,^3^,^4^)**^, including plain radiography (X-ray), ultrasonography, computed tomography, and magnetic resonance imaging (MRI). In general, MRI is considered to be the most effective method for diagnosing apophysitis. Although less commonly employed, ultrasonography has also been shown to be effective for diagnosing apophysitis^**(^5^)**^. Although MRI provides the most accurate information for both types of injuries, its high cost, longer acquisition time, limited availability, and contraindications often restrict its routine use^**(^3^)**^. Conversely, X-ray is widely accessible, cost-effective, and recommended for the initial assessment of trauma. Power Doppler, a tool commonly used in rheumatology to evaluate inflammatory processes^**(^6^)**^, is still underutilized in sports medicine^**(^2^,^7^,^8^)**^.

Given these considerations, the primary objective of this study was to determine the diagnostic accuracy of X-ray in detecting apophyseal avulsion fractures in adolescent soccer players. Secondary objectives included investigating potential associations among findings from X-ray, MRI, and power Doppler, with a focus on identifying alterations that may influence diagnosis and clinical management.

## MATERIALS AND METHODS

This was an observational cross-sectional study involving youth athletes from Vasco da Gama Sociedade Anônima do Futebol, a prominent soccer academy in the city of Rio de Janeiro, Brazil. Due to the exploratory nature of the study and ease of access to participants, a convenience sample was selected. The study was approved by the Research Ethics Committee of the Hospital Universitário Pedro Ernesto (HUPE), operated by the Universidade do Estado do Rio de Janeiro (UERJ), under protocol number 56309422.7.0000.5259. Informed consent was obtained from the parents or legal guardians of all participants.

The following inclusion criteria were applied: being a male athlete between 9 and 17 years of age; and being under clinical suspicion of apophyseal avulsion fracture, characterized by acute pain during sports activity, tenderness upon palpation, swelling, pain with active contraction or passive stretching, and functional limitation. Athletes meeting those criteria were referred to the imaging department at HUPE-UERJ within the first 24 h after the trauma for X-ray, power Doppler, and MRI. Those diagnosed with muscle strain via MRI were excluded from the study.

All X-rays were obtained with a conventional radiography system (Radspeed MC; Shimadzu Corporation, Tokyo, Japan) in anteroposterior and oblique panoramic views of the pelvis. Avulsion fracture was diagnosed if apophyseal displacement was observed in any view.

Power Doppler examinations were conducted with a dedicated ultrasound system (Logiq E10; GE Healthcare, Chicago, IL, USA) using 14-MHz linear and 20-MHz hockey stick transducers. The thigh was scanned in the transverse and longitudinal planes: anteriorly, from the iliac crest to the quadriceps insertion; and posteriorly, from the ischial tuberosity to the popliteal fossa. Examination parameters included a pulse repetition frequency of 750 Hz, a low wall filter, and gain adjustments to minimize artifacts. The identification of abnormal vascular flow (any asymmetric vascular signal compared to the contralateral side) in the apophysis or periphyseal tissues was considered a positive finding on power Doppler.

The MRI scans of the thighs were performed in a 1.5-T scanner (Optima MR 360; GE Healthcare), employing T1-weighted and short-tau inversion recovery sequences in the axial and coronal planes, without the use of intravenous contrast. The MRI criteria for diagnosing avulsion fracture included apophyseal displacement, bone edema, physeal hyperintensity, periphyseal edema, and intermuscular fluid collections. Bone and periphyseal edema without displacement was deemed indicative of apophysitis.

Six radiologists, each with more than ten years of experience, evaluated the X-ray, MRI, and power Doppler studies, with two radiologists being assigned to each imaging modality. Interobserver and intraobserver agreement were assessed for avulsion fracture and apophyseal vascular hyperemia.

Inferential statistical analysis was conducted by using Pearson’s chi-square test to assess the associations between imaging findings obtained in X-ray, MRI, and power Doppler examinations. A significance level of *p* < 0.05 was adopted for all tests. The diagnostic accuracy of X-ray was evaluated by using MRI as the reference standard. The calculations included sensitivity, specificity, positive predictive value, negative predictive value, and overall accuracy, which were derived from a 2×2 contingency table. Cohen’s kappa coefficient (κ) was calculated to assess interobserver and intraobserver agreement in interpreting the X-ray, MRI, and power Doppler images. Corresponding *p*-values and 95% confidence intervals were also calculated to determine whether the level of agreement was statistically significant, with the significance level for κ also set at *p* < 0.05. All analyses were performed with the IBM SPSS Statistics software package, version 26.0 (IBM Corp., Armonk, NY, USA).

## RESULTS

### Descriptive analysis

**Figure 1:**
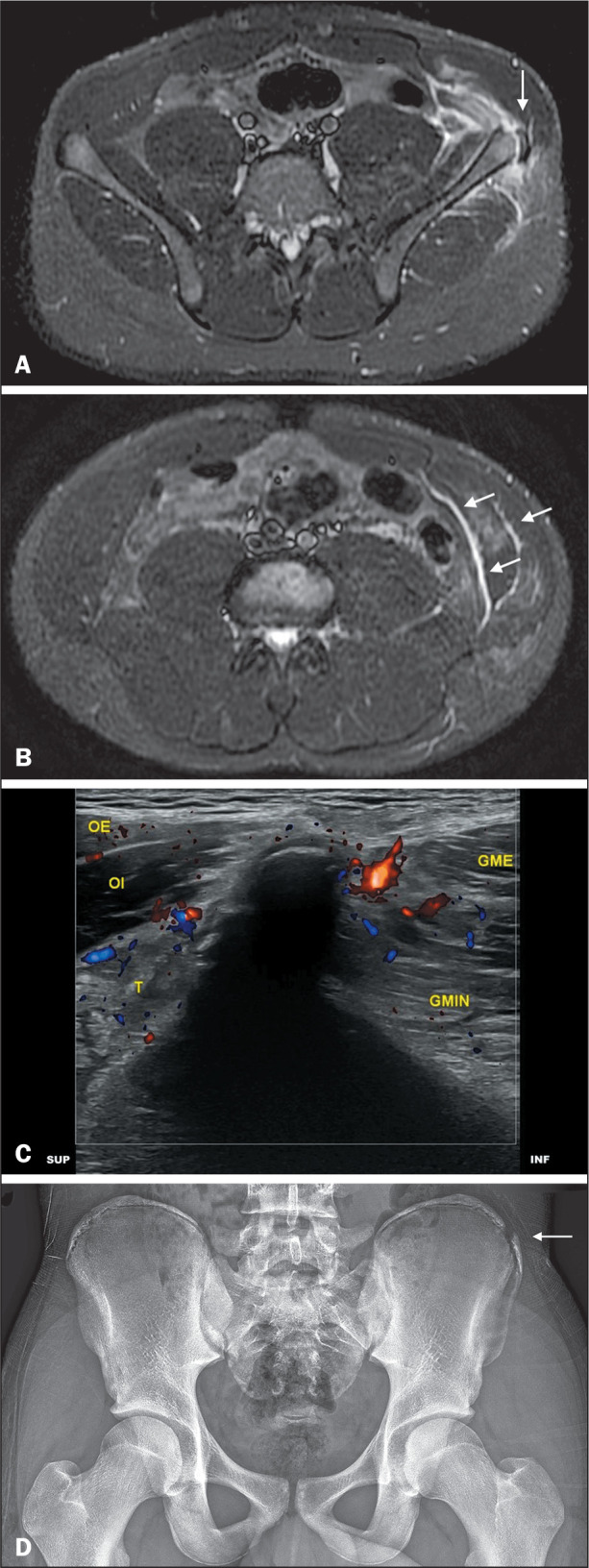
Avulsion fracture. Thirteen-year-old athlete with anterior left thigh pain. A: Axial short-tau inversion recovery MRI of the thighs showing avulsion of the left iliac crest (arrow), together with periphyseal edema and fluid collections. B: Axial short-tau inversion recovery MRI of the thighs showing fluid collections between the muscular layers of the lateral abdominal wall (arrows). C: Sagittal ultrasound view of the iliac crest with power Doppler showing periphyseal vascular hyperemia. D: Anteroposterior panoramic X-ray of the pelvis showing avulsion of the left iliac crest apophysis (arrow).

GME, gluteus medius; GMIN, gluteus minimus; INF, inferior; EO, external oblique; IO, internal oblique; SUP, superior; T, transversus abdominis.

**Figure 2:**
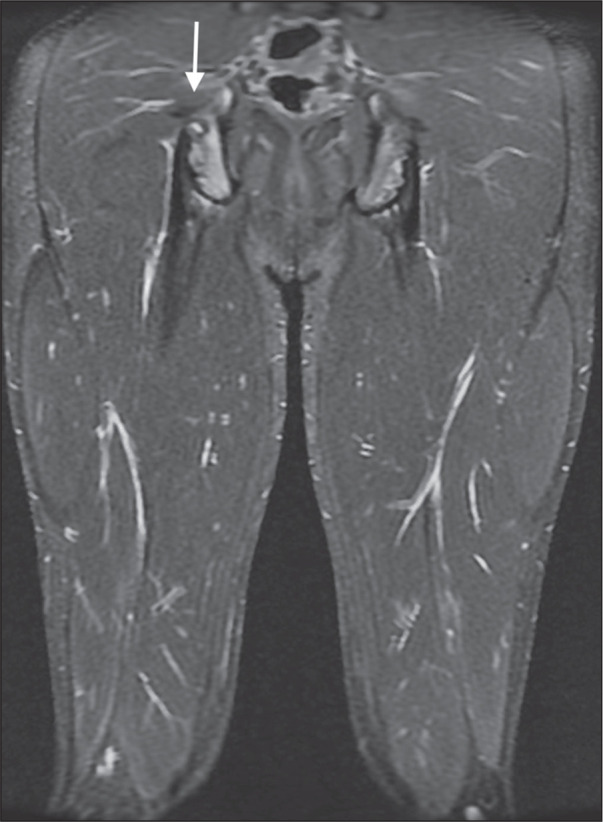
Apophysitis. Fifteen-year-old athlete with right gluteal discomfort. Coronal short-tau inversion recovery MRI of the thighs showing bone marrow edema of the ischial tuberosity apophysis (arrow).

**Figure 3:**
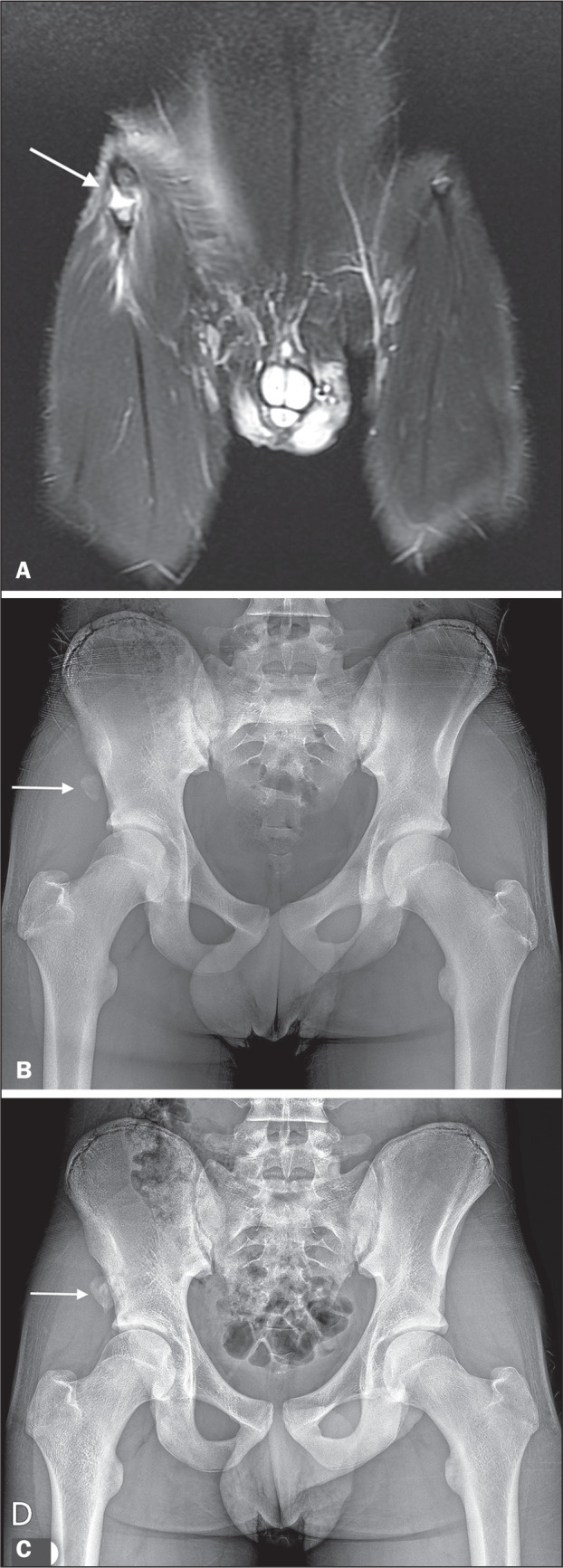
Avulsion fracture. Fourteen-year-old athlete with anterior left thigh pain. A: Coronal short-tau inversion recovery MRI of the thighs showing avulsion of the right anterior inferior iliac spine (arrow). B: Anteroposterior panoramic X-ray of the pelvis showing an avulsed fragment of the right anterior inferior iliac spine (arrow). C: Anteroposterior panoramic pelvic X-ray obtained six months after the trauma, showing persistence of the avulsed fragment (arrow).

The study sample comprised 33 male athletes who underwent MRI scans. The mean age of the participants was 13.1 ± 1.7 years. Suspected injuries to the right lower limb were reported in 17 athletes (51.5%). The most commonly affected apophyses, in descending order, were the anterior inferior iliac spine, in 14 cases (42.4%), the ischial tuberosity, in 12 (36.4%), the iliac crest, in three (9.1%), the anterior superior iliac spine, in three (9.1%), and the lesser trochanter of the femur, in one (3.0%). Figures 1, 2, and 3 show exemplary images of the injuries.

The MRI scans revealed avulsion injuries in 21 athletes (63.6%) and apophysitis in 11 (33.3%). Only one athlete (3.0%) had normal MRI findings. The MRI findings for the cases of avulsion fracture included apophyseal displacement in all 21 cases (100%), periphyseal edema in 20 cases (95.2%) (Figures 1A and 3A), bone marrow edema in six cases (28.6%), and intermuscular fluid collections in 14 cases (66.7%) (Figure 1B). In cases of apophysitis, periphyseal edema was observed in five cases (45.5%). As illustrated in Figure 2, bone marrow edema was noted in all 11 cases of apophysitis (100%). Intermuscular fluid collections were identified in only two (9.5%) of the 21 athletes with avulsion fractures.

In the cases of avulsion fracture, the median displacement was 3.3 mm (range, 1.8-6.6 mm). Of the 21 avulsion fracture cases, 17 (81.0%) exhibited a predominance of periphyseal edema. In contrast, 10 (90.9%) of the 11 cases of apophysitis showed a predominance of bone marrow edema.

Power Doppler examinations were conducted in 31 athletes, showing vascular hyperemia, as shown in Figure 1C, in 10 cases (32.3%).

A total of 25 athletes underwent X-ray, which identifying avulsion fracture, as depicted in Figures 1D, 3B, and 3C, in 11 cases (44.0%). The median displacement observed on X-ray was 4.7 mm (range, 2.5-7.5 mm).

### Reproducibility of X-ray, Doppler, and MRI readings

Each reader (two per imaging modality) evaluated the X-ray, power Doppler, and MRI examinations performed at two time points: immediately after and two weeks after the trauma. For the X-ray and MRI examinations, intraobserver and interobserver agreement was assessed for the diagnosis of avulsion fracture. For power Doppler, intraobserver and interobserver agreement was assessed for flow detection.

For the X-ray examinations, the level of intraobserver agreement was almost perfect (κ = 0.85) for reader 1 and substantial (κ = 0.72) for reader 2. For those same examinations, the level of interobserver agreement was almost perfect (κ = 0.85).

For the power Doppler examinations, the level of intraobserver agreement was perfect (κ = 1.00) for reader 1 and almost perfect (κ = 0.92) for reader 2. For those same examinations, the level of interobserver agreement was almost perfect (κ = 0.93).

For the MRI examinations, the level of intraobserver agreement was perfect (κ = 1.00) for reader 1 and substantial (κ = 0.63) for reader 2. For those same examinations, the level of interobserver agreement was moderate (κ = 0.53).

### Accuracy of X-ray

A total of 25 athletes underwent X-ray and MRI. The accuracy of X-ray was evaluated against MRI, which was considered the gold standard for comparison. X-ray showed moderate accuracy compared with MRI for detecting apophyseal avulsion fractures, with high specificity and positive predictive value (Table 1).

**Table 1:** Comparison of X-ray and MRI for diagnosing avulsion injuries.

Avulsion on X-ray, n	Avulsion on MRI, n		
	Yes	No	Total
Yes	9	2	11
No	9	5	14
Total	18	7	25

Sensitivity: 50%; Specificity: 71.4%; Positive predictive value: 81.8%; Negative predictive value: 35.7%; Accuracy: 56%.

### Associations between power Doppler and avulsion fracture on X-ray and MRI

The evaluation of the relationship between the power Doppler and X-ray findings revealed no significant association, suggesting that increased vascular flow does not necessarily correlate with radiographic changes (Table 2). Conversely, a significant association was observed between positive power Doppler and avulsion fracture confirmed by MRI, reinforcing the hypothesis that local hyperemia is a marker of acute traction on the apophysis (Table 3).

**Table 2:** Association between power Doppler results and avulsion fracture on X-ray.

Power Doppler result, n	Avulsion fracture on X-ray, n	
	Yes	No
Positive	6	3
Negative	4	10
Total	10	13

Chi-square test (χ^^2^^) = 1.87; *p* = 0.171.

**Table 3:** Association between power Doppler and avulsion fracture on MRI.

Power Doppler result, n	Avulsion fracture on MRI, n	
	Yes	No
Positive	10	3
Negative	10	11
Total	20	11

Chi-square test (χ^^2^^) = 5.99; *p* = 0.0144.

### Associations that avulsion fracture and apophysitis showed with periphyseal edema, intermuscular fluid collections, and bone marrow edema on MRI

On MRI, avulsion fractures showed a strong association with periphyseal edema and intermuscular fluid collections, whereas bone marrow edema was typically absent in those cases (Table 4). For apophysitis, the opposite pattern was observed: a predominance of bone marrow edema, often isolated and without signs of periphyseal edema or intermuscular fluid collection (Table 5).

**Table 4:** Associations between avulsion fracture and MRI findings.

MRI finding	Avulsion fracture, n		x^^2^^	*P*
	Yes	No		
Periphyseal edema, n				
Yes	20	5	9.19	0.0024
No	1	7		
Intermuscular fluid collection, n				
Yes	14	1	8.26	0.0040
No	7	11		
Bone marrow edema, n				
Yes	6	11	9.78	0.0018
No	15	1		

**Table 5:** Associations between apophysitis and MRI findings.

MRI finding	Apophysitis, n			*P*
	Yes	No		
Periphyseal edema, n				
Yes	6	19	4.79	0.0287
No	6	2		
Intermuscular fluid collection, n				
Yes	2	14	5.77	0.0163
No	10	7		
Bone marrow edema, n				
Yes	12	0	14.83	0.00012
No	5	16		

## DISCUSSION

Initially, indicators of interobserver and intraobserver agreement were determined for the diagnosis of avulsion fracture and for power Doppler flow readings. The level of agreement was found to range from moderate to excellent. The locations of avulsion fracture, predominantly at the anterior inferior iliac spine and ischial tuberosity, align with previously reported findings^**(^1^,^4^,^5^)**^.

Despite its relevance in sports medicine, the topic of avulsion fractures remains underexplored in the radiology literature^**(^9^-^11^)**^. Our approach can be divided into three main foci: the importance of X-ray in diagnosing sports-related avulsion fractures; the role of power Doppler in assessing sports-related trauma; and MRI findings that help differentiate between avulsion fractures and apophysitis.

We found that X-ray demonstrated moderate performance in diagnosing avulsion fractures. The high positive predictive value suggests that when an avulsion fracture is detected on X-ray, the diagnosis is likely accurate. Its high specificity further reinforces the utility of X-ray in confirming positive cases. However, the low sensitivity and negative predictive value indicate that many avulsion fractures go undetected on X-ray, making MRI necessary for reliably excluding the injury. Therefore, X-ray is effective as a confirmatory test but not reliable as a screening tool. In patients with a strong clinical suspicion, a negative X-ray should not rule out the possibility of an avulsion fracture. This finding contrasts with the conclusions of Cirimele et al.^**(^4^)**^ and Albtoush et al.^**(^12^)**^ , who argued that clinical examination and X-ray were sufficient for diagnosing avulsion fractures. Whereas those authors highlighted tissue overlap as the main limitation of X-ray, the present study identified two additional factors: the inability of X-ray to identify minimal apophyseal fragment displacement and its inability to detect periphyseal changes. Because our sample included patients with small avulsion displacements, X-ray struggled to identify them. In addition, findings such as periphyseal edema and intermuscular fluid collections-which are important for confirming an avulsion fracture-are not visible on X-rays.

To our knowledge, this is the first study to statistically evaluate the role of power Doppler in diagnosing avulsion fractures. During the formation of repair tissue, neoangiogenesis commonly occurs and can be detected by power Doppler, depending on the severity and timing of the trauma^**(^13^-^15^)**^. Previous studies have reported vascular hyperemia at fracture sites, such as those associated with stress fractures^**(^16^)**^. In our study, we found an association between abnormal power Doppler vascular flow and avulsion fracture detected by MRI; all 10 cases with vascular hyperemia also had avulsion fractures. Conversely, the absence of vascular flow was nonspecific, occurring at similar rates in the avulsion and non-avulsion groups. There was no significant association between power Doppler positivity and avulsion fracture identified by X-ray, although a larger sample may be needed in order to confirm this finding.

Differentiating between avulsion fracture and apophysitis on MRI can be challenging, especially when there is no visible displacement. However, certain MRI findings were helpful: avulsion fractures were associated with periphyseal edema and intermuscular fluid collections, whereas apophysitis was predominantly associated with bone marrow edema. Since apophysitis results from low-intensity repetitive trauma that does not typically cause discontinuity, significant periphyseal soft tissue changes are likely absent. The cartilage-bone complex functions like an enthesis organ, in which bone marrow edema is a secondary manifestation of chronic overload at the enthesis. This phenomenon, described in enthesitis, was extensively discussed years ago by Benjamin et al.^**(^17^,^18^)**^ and Shaw et al.^**(^19^)**^. In contrast, avulsion fractures result from acute, high-impact trauma, which explains the presence of periphyseal edema and intermuscular fluid collections. Because the mechanism does not involve chronic enthesis overload, bone marrow edema tends to be absent or minimal-except in cases with direct enthesis involvement.

Our study has some limitations. First, the sample was small and there was no sample size calculation. In addition, there was an unequal distribution of imaging modalities, which limited comparisons among equal numbers of athletes across methods. Future studies should evaluate the accuracy of B-mode ultrasound, alone or in combination with X-ray, as a lower-cost and more accessible alternative to MRI.

In conclusion, X-ray proved to be a reliable method for confirming avulsion fractures but not for ruling them out. Power Doppler was significantly associated with avulsion fractures. Avulsion fractures were associated with intermuscular fluid collections and periphyseal edema, whereas apophysitis was associated with bone marrow edema. Together, these MRI findings significantly aid in the differential diagnosis between avulsion fractures and apophysitis.

## Data Availability

Data sets related to this article will be available upon request to the corresponding author.
